# Single nucleotide polymorphism discovery from expressed sequence tags in the waterflea *Daphnia magna*

**DOI:** 10.1186/1471-2164-12-309

**Published:** 2011-06-13

**Authors:** Luisa Orsini, Mieke Jansen, Erika L Souche, Sarah Geldof, Luc De Meester

**Affiliations:** 1 Laboratory of Aquatic Ecology and Evolutionary Biology, K.U. Leuven, Ch. Deberiotstraat 32, 3000 Leuven, Belgium; 2 Laboratory of Animal Diversity and Systematics, K.U. Leuven, Ch. Deberiotstraat 32, 3000 Leuven, Belgium; 3 Institut Pasteur, Plate-Forme Intégration et Analyse Génomiques, 28 Rue du Docteur Roux, 75724 Paris Cedex 15, France

## Abstract

**Background:**

*Daphnia *(Crustacea: Cladocera) plays a central role in standing aquatic ecosystems, has a well known ecology and is widely used in population studies and environmental risk assessments. *Daphnia magna *is, especially in Europe, intensively used to study stress responses of natural populations to pollutants, climate change, and antagonistic interactions with predators and parasites, which have all been demonstrated to induce micro-evolutionary and adaptive responses. Although its ecology and evolutionary biology is intensively studied, little is known on the functional genomics underpinning of phenotypic responses to environmental stressors. The aim of the present study was to find genes expressed in presence of environmental stressors, and target such genes for single nucleotide polymorphic (SNP) marker development.

**Results:**

We developed three expressed sequence tag (EST) libraries using clonal lineages of *D. magna *exposed to ecological stressors, namely fish predation, parasite infection and pesticide exposure. We used these newly developed ESTs and other *Daphnia *ESTs retrieved from NCBI GeneBank to mine for SNP markers targeting synonymous as well as non synonymous genetic variation. We validate the developed SNPs in six natural populations of *D. magna *distributed at regional scale.

**Conclusions:**

A large proportion (47%) of the produced ESTs are *Daphnia *lineage specific genes, which are potentially involved in responses to environmental stress rather than to general cellular functions and metabolic activities, or reflect the arthropod's aquatic lifestyle. The characterization of genes expressed under stress and the validation of their SNPs for population genetic study is important for identifying ecologically responsive genes in *D. magna*.

## Background

Single Nucleotide Polymorphisms (SNPs), defined as single-base changes or short insertion or deletion mutations (indels), are the most abundant class of genetic variation found in eukaryotic genomes. SNPs are widespread, and present in both coding and non coding regions [1-3]. Until few years ago, the use of SNP markers was limited to model organisms with sequenced genomes, mostly because of the costs associated with SNP discovery. Methods for indirect SNP discovery detect heteroduplexes on the basis of mismatch-induced altered DNA characteristics [4-8], whereas typical direct SNP discovery strategies [9,10] involve sequencing of locus-specific amplification (LSA) products from multiple individuals. One of the most common strategies adopted for SNP development in non-model organisms is the use of Expressed Sequence Tags (ESTs) as a resource for SNP marker detection [11-14]. This is a consequence of the increasing availability of EST libraries for non-model organisms. As a result, SNPs are becoming increasingly important for research on non-model organisms. SNPs offer the potential for genome wide scans of selectively neutral as well as adaptive variation [15,16], with simple mutation models and powerful analytical methods [17], and with application to noninvasive analysis and historical DNA [18].

Most genetic surveys of natural populations focus on neutral loci. Whereas this provides valuable insights into the historical demography and evolution of populations (see [2]), it does not allow to understand the dynamics of genes that affect fitness along environmental gradients. *Daphnia *(Crustacea: Cladocera) offers a unique opportunity to study neutral and selective variation in natural populations with a known ecological background. *Daphnia *play a pivotal role in the ecology of standing waters, are widely used in population studies and environmental risk assessments, and are supported by a large community of ecologists, evolutionary biologists and ecotoxicologists [19-21]. This is in part due to their ease of culture, convenient size, short generation time and cyclic parthenogenetic reproduction, which make them very suitable for laboratory and field experiments, experimental evolution, and quantitative genetic analyses in multiple environments. Thanks to the sustained efforts of the Daphnia Genomics Consortium (http://daphnia.cgb.indiana.edu, DGC), *Daphnia *is also regarded as a model invertebrate in ecological genomics [21,22]. *Daphnia magna *is, especially in Europe, intensively used to study stress responses to pollutants, climate change, and antagonistic interactions with predators and parasites [23-25]. *Daphnia *has also been subject to intensive population genetic study, with a strong focus on the impact of its peculiar reproduction mode, cyclical parthenogenesis, population genetic structure and among-population genetic differentiation [26-29]. Yet, although some knowledge has recently been acquired in the fields of functional responses to parasite infection [30] and proteomics [31], precious little is known about the complex interaction between neutral genetic variation, reflecting population genetic structure and demography, and the functional genomics underpinning phenotypic responses to environmental stressors. Among other reasons, the lack of suitable markers for functional traits has been one of the main limitations.

We developed three EST libraries using clonal lineages of *D. magna *exposed to standardized selection pressures, namely fish predation, exposure to parasites (*Pasteuria ramosa*), and exposure to pesticides (carbaryl). These environmental stressors are known to induce pronounced micro-evolutionary responses in *D. magna *[23-25]. Our EST sequences as well as EST sequences published in NCBI at the time of the analysis were mined for SNP markers targeting synonymous and non-synonymous polymorphisms. An *in silico *discovery tool purposely designed to mine EST sequences (Souche et al, in prep) was used for SNP discovery. The newly developed SNPs were validated by genotyping individuals from six natural populations of *D. magna *distributed at regional scale. The identification and characterization of genes differentially expressed in stress conditions and the validation of SNP mutations that could be linked to specific environmental stressors opens new interesting perspectives in the study of functional polymorphism in natural populations of *D. magna*.

## Methods

### Development of EST libraries

#### Exposure of clones to environmental stressors

The clones used to develop the EST libraries were two genetic isolates (M10 and Mu11) exposed independently to three environmental stressors that are known to induce micro-evolutionary responses in *D. magna*: fish predation, parasite infection and pesticide exposure. Clone M10 was hatched from Oud-Heverlee pond in Belgium (see [24] for information on habitat), whereas clone Mu11 was isolated from a pond in Germany (see [32]). Animals were grown for two generations under standard light/food regime to eliminate residual maternal effects after hatching. Clone Mu11 was exposed to fish kairomones enriched medium, while clone M10 was exposed independently to parasites and pesticides. All exposed animals were grown in a climate chamber (20+/-1°C) in 100 ml ADaM medium [33] and daily fed with 1 × 10^5 ^cells of *Scenedesmus obliquus*/ml. Exposure to the environmental stressors was performed on juveniles not older than 24 h released from the second clutch. Juveniles were exposed in a concentration of 10 animals/50 ml, daily fed with 1 × 10^8 ^cells of *S. obliquus*/ml, and kept in daily refreshed medium.

The exposure to fish predation was mimicked by culturing *D. magna *in fish kairomone enriched ADaM medium [33]. The medium was obtained by filtering (0.450 μm mesh) and diluting water (5 times) from a 20L aquarium where three gold orfes (*Leuciscus idus*) were kept for 24 h.

The common endoparasite of *D. magna, Pasteuria ramosa*, was used to induce parasite infection. *P. ramosa *infection happens via horizontal transmission of spores released from dead infected hosts. Monoclonal cultures of the clone M10 were exposed to a spore solution consisting of squashed and filtered (60 μm nylon filter) infected animals. A final concentration of 1 × 10^6 ^mature spores/ml was used to infect the *Daphnia *host. To avoid that adaptation to specific strains of the parasite spores would affect the response to infection, the host and the spores were collected from two distinct ponds in Belgium [hosts were hatched from Oud-Heverlee pond, whereas *P. ramosa *spores were collected from the sediment of OM2 pond (for details on the ponds see [24]). A detailed description of the infection method is reported elsewhere [34].

We used carbaryl (1-napthyl methylcarbarmate), a commonly used pesticides in agriculture, as a model pesticide. Different concentrations of carbaryl (CAS 63-25-2, purity 99.8%, Sigma-Aldrich, Germany) were used: 5.6 μg/L, 8.0 μg/L and 11.4 μg/L. These concentrations were determined by earlier work on pesticide exposure to be sublethal but at the same time affect *Daphnia *fitness [35,36].

After exposure, animals were transferred to liquid nitrogen for storage. Juveniles exposed to fish predation and parasite infection were stored after 48 h and 96 h. Carbaryl exposed animals were stored after 48 h, 96 h and 144 h exposure time. Depending on the age and the survival of the exposed *Daphnia*, between 18 and 53 individuals were pooled for RNA extraction.

#### Construction of cDNA libraries and sequencing of ESTs

Total RNA was extracted from the pool of exposed and non-exposed (control) samples using the Trizol^® ^extraction method (Invitrogen, Life technologies, Belgium), following the manufacturer's instructions. After extraction and DNAse treatment (Fermentas, Germany; [37]), RNA purity was checked with a spectrophotometer (NanoDrop Technologies, USA).

Pooled RNA samples (three time points -48, 96 and 144 h- for the carbaryl exposures and two -48 and 96 h- for parasite and fish kairomone exposures) from individual stressors were used to create three separate libraries. Each library represents the genes differentially expressed in a pool of exposed individuals versus a control (non exposed individuals). The construction of the subtractive libraries was done according to Soetaert et al. [38]. In brief, we used a combination of the SMART™ PCR cDNA synthesis kit (Clontech, USA) and the PCR-Select™ cDNA subtraction kit (Clontech, USA) to obtain libraries by means of Suppression Subtractive Hybridization [39]. Differently expressed genes in the three subtraction libraries were cloned using the pGEM^®^-T Easy Vector System II (Promega, USA) following the manufacturer's instructions. The gene fragments were PCR amplified with the cloning vectors, M13F (5' CGA CTG TGT AAA ACG ACG GCC AG 3') and M13R (5'CAG GAA ACA GCT ATG ACC ATG ATT ACG CC 3'). The PCR products were purified with Exo-SAP (Fermentas, USA) at a concentration of 10 u/5 μl of PCR product prior sequencing. PCR products were single-strand sequenced using Big Dye terminator chemistry on a CEQ™ 8000 automated sequencer (Beckman Coulter). The PCR fragments were sequenced with the vector primers SP6 (5' ATT TAG GTG ACA CTA TAG 3') and T7 (5' TAA TAC GAC TCA CTA TAG GG 3). Sequence assembly and editing were done using CodonCode Aligner http://www.codoncode.com.

#### EST assembly, annotation and processing for SNP development

cDNA fragments were trimmed from residual vector sequences using CodonCode Aligner. Low quality and short sequences fragments (fragments <50 bp) were removed from the total set of sequences. Base calling and attribution of quality values were performed using Phred [40], using default parameters. The software cross_match (http://www.phrap.org/phredphrapconsed.html#block_phrap) was used to mask oligonucleotides, primers and adapters used during the library construction on the remaining ESTs. Masked sequences, polyA tails and low complexity sequences were trimmed with SeqClean using default parameters and a minimum accepted length of 50bp (http://compbio.dfci.harvard.edu/tgi/software/). The trimmed fragments and EST sequences obtained from NCBI GenBank at the time of the analysis [41] (Additional file 1) were assembled in clusters based on their similarities using TGICL [42] and default parameters, except for the overlap percent identity cut-off that was set to 93 instead of 80. A quality value of 20 was arbitrarily assigned to all EST nucleotides downloaded from NCBI GenBank in order to process sequences with unknown quality in the SNP pipeline (see below). The group of ESTs assembled in one consensus sequence and the remaining unclustered ESTs (referred hereafter as contigs and singlets) were used for sequence similarity searches against the NCBI and the http://wfleabase.org/ [19] genome databases using Blast [43]. These contigs and singlets were subsequently annotated using Open Reading Frame (ORF) prediction by OrfPredictor [44], Gene Ontology assignments by Blast2GO [45] and Prosite functional protein domains by ScanProsite [46]. Orthologs and paralogs were identified using the OrthoMCL database that includes 1,270,853 sequences from 138 genomes [47].

### SNP markers development

The clusters obtained by assembling EST sequences from GenBank and our ESTs were mined for SNPs using a pipeline that integrates six freely available SNP discovery tools: SNPserver [48], PolyBayes [49], Quality SNP [50], PolyFreq [51], MiraEST [52], and ssahaSNP [53]. Stringent quality criteria were used for SNP mining. We excluded clusters containing singlet sequences for obvious reasons; we considered only one contig per cluster when multiple contigs were present to avoid redundancy; a mismatch was regarded as SNP candidate if it appeared at least twice in the alignment. Consequently, only the contigs containing four overlapping ESTs were searched for SNPs. The ACE file produced by TGICL was used as input of the pipeline. We also designed SNPs probes and PCR primers for five nuclear genes, including one nuclear receptor gene [Ultraspiracle (Usp)] and four metabolic enzyme genes [Phosphoglucose isomerase (Pgi), Mannose-phosphate isomerase (Mpi), l-lactate dehydrogenase (Ldh), Enolase (Enol)]. These genes were previously used in a study involving *D. magna *and *D. pulex *[54]. The sequences were kindly provided by Christoph R. Haag.

### SNP validation and characterization

SNP loci were validated by genotyping natural populations of *D. magna*. We sampled the surface sediment layer of six populations (185 dormant eggs) of *D. magna*, scattered across Belgium (three from the coast and three from the inland, Additional file 2). By hatching dormant eggs from the superficial sediment layers, we ensured to capture the genetic diversity of the populations without it being affected by clonal erosion typically occurring in the active populations of cyclical parthenogenetic species [55,56]. Our approach aims at sampling genetic diversity as it occurs at hatching, at the beginning of the growing season. Genotyping of SNPs was performed using the MassARRAY platform from Sequenom (San Diego, USA) at the VIB Vesalius Research Center, Leuven, Belgium. Genomic positions of the genetic variants were selected and 70 bp-up and downstream sequences were used for primer design with the Sequenom MassARRAY Assay Design 3.1 software with default parameters. Respective forward PCR, reverse PCR and extension primers for the Sequenom genotyping assays can be found in supplementary material (Additional file 3). Multiplexes levels were between 17 and 40. The genotyping was performed according to the iPLEX protocol from Sequenom (available at http://www.sequenom.com/). Quality control criteria were adopted (water as negative control and inter-plate duplicates testing).

SNP markers were characterized by counting the number of synonymous and non-synonymous mutations, and by identifying the codon position responsible for the non-synonymous changes.

### Population genetics analysis

A test for outlier SNPs was performed to identify the strictly neutral SNPs to use in population genetic statistics in order to validate the newly developed markers. The outlier loci were identified with two methods, Lositan [57] (a selection detection workbench constructed around the program fdist [58]) and BAYESCAN [59]. The simulation conditions were as follows. For Lositan [57], we used 10,000 simulation replicates (infinite allele model, IAM) for a sample size of 50 individuals, with forced mean F_ST _calculated on the real data set. Multiple runs (generally 3) were run to avoid spurious results. For BAYESCAN, 10 pilot runs of 5,000 iterations and an additional burn-in of 50,000 iterations were followed by 100,000 iterations (sample size of 5,000 and thinning interval of 20) to identify loci under selection from locus specific Bayes factors. A Bayes factor of 3, corresponding to a posterior probability of 0.5 (substantial selection) was considered as the minimal threshold for a locus to be considered under selection. Multiple runs (generally 3) were run to avoid spurious results.

The strictly neutral loci were used to estimate the among-population differentiation with the Microsatellite Analyser (MSA) software v 3.12 [60]. They were also used in an analysis of population genetic structure and inference of population demography. The spatial genetic structure was described using the Bayesian analysis implemented in the BAPS software [61-63]. We performed a non-spatial genetic mixture analysis using individuals and populations as basic units to be clustered. Different starting values of K (2 to 12) were used to verify the robustness of the results. A Mantel test (10,000 permutations) was performed using IBD online software [64] to test for Isolation by Distance.

## Results

### EST annotation

The three EST libraries yielded 1,698 gene fragments. Of these fragments, 480 were from the parasite library, 480 from the fish library and 738 from the pesticide library. After a quality trimming, 1,070 fragments remained (301 from the fish library, 366 from the parasite library and 403 were from the pesticide library; GenBank accession numbers: HO045245-HO046616). Of the total gene fragments, 685 showed significant similarity (Blast e < 10^-5^) with genes within the NCBI non-redundant protein database (64% of the total sequenced fragments, Additional file 4). Of these, 4 matched nuclear eukaryotic genes, 16 matched mitochondrial genes, 82 matched ribosomal genes, and 196 showed homology to known proteins in other organisms (Additional file 4). An additional 60 ESTs aligned to annotated *D. pulex *and *D. magna *genes, and 327 ESTs aligned to predicted or conserved hypothetical proteins. Of the 685 gene fragments, 287 have a gene prediction based on homology with known genes in other organism. The annotated gene fragments showed significant similarity with a number of species, including a large proportion of insects (65%) (Additional file 5).

### Annotation of assembled ESTs

The sequences considered for SNP mining, including GeneBank and our sequences for assembling into a non-redundant unigene set had an average length of 454 bp and a standard deviation of 137 bp. Over 80% of all ESTs were longer than 350 bp. From the total number of ESTs, 1,674 clusters of sequences were produced. A total of 10,737 ESTs, representing 75.3% of the 14,253 processed ESTs, were assembled in 1,812 contigs, with a redundancy of 75.3%. Only 2,446 ESTs (17.2% of processed ESTs) remained singletons. Of the total number of obtained contigs, 878 (48.5%) contained 2 ESTs and 143 (7.9%) contained more than 10 ESTs. Out of the 1,812 contigs, 55 (3.0%) contained only ESTs produced in this study, 1,483 (81.9%) contained only ESTs downloaded from GenBank, and 274 (15.1%) contained a mix of both ESTs. 1,183 (65%) of these contigs showed significant sequence similarity (Blast e < 10^-5^) with genes within the NCBI non-redundant protein database. An additional 421 contigs were aligned to annotated *D. pulex *proteins. Therefore, 88.5% of the chosen contigs are given putative functions based on sequence similarity to annotated proteins from genome of other species; 26% are unique to the lineage leading to *Daphnia*. The 574 contigs containing more than 4 overlapping sequences were mined for SNPs: 414 (72%) showed significant sequence similarity with genes within NCBI, 122 additional contigs were aligned to annotated *D. pulex *proteins. Therefore, 93% of the chosen contigs are given putative functional annotations; 23% are unique to the lineage leading to *Daphnia*.

### *In vitro *development of SNP markers

The contigs containing more than 4 overlapping sequences were mined for SNPs. The discovery tools used in the pipeline identified different numbers of SNPs (AutoSNP: 778; PolyBayes: 757, QualitySNP: 641, PolyFreq: 51; MiraEST: 333) depending on the specific quality parameters. The number of SNP candidates detected per polymorphic contig ranged between 27 and 1, with 96 contigs showing only one candidate SNP, 163 contigs showing between 2 and 9 candidate SNPs and 17 contigs showing more than 10 candidate SNPs. A total of 986 candidate SNPs were discovered. Excluding the candidate SNPs that were contiguous, close to an indel or to the 5' and 3' ends of the ESTs, and the ones that presented high polymorphism in the flanking regions, we had 159 candidate SNPs left, including more than one candidate per EST in some contigs. PCR primers and oligonucleotide probes were designed only for SNPs indentified by a minimum of 3 discovery tools, in order to avoid false positives. Moreover, we targeted only one SNP per contig to use in subsequent genotyping optimization assay. The use of a single candidate SNP per EST was chosen to avoid complications in the subsequent population genetic analyses caused by linkage among SNPs within the same sequence. Excluding the unsuitable candidates based on all the criteria outlined above, we tested 138 SNPs with Sequenom.

### SNP validation and characterization

**Figure 1:**
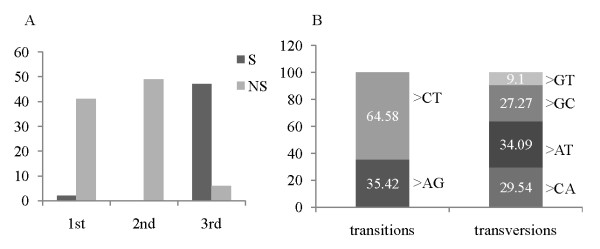
**Synonymous and non-synonymous substitutions in the EST linked SNPs**. (A) Synonymous (S) and non-synonymous (NS) mutations in the set of SNPs developed in this study at the three codon positions. (B) Proportion of transitions and transversions in the developed SNPs.

A total of 147 SNP markers (including EST linked SNPs and markers developed from the nuclear genes) were designed and arranged in five assays for SNP typing with Sequenom (Additional file 3). Seven of the 147 markers could not fit in any assay design, therefore were dropped from further analysis (PCR primers and SNP probes not reported). Of the total set of designed SNPs, 43 (29.25%) were on the first, 49 (33.33%) on the second and 54 (36.73%) on the third codon position. Fifty one single point mutations were synonymous (S) whereas 96 were non-synonymous (NS). The majority of NS mutations was located on the first and second codon position. Most of the point mutations on the third codon position were synonymous; only 11% was NS (Figure 1A, Additional file 6). Information on the protein changes at the NS sites is reported in Additional file 6. The SNPs consist mostly of transitions. The most common transition is C↔T. The most frequent transversion is A↔T (Figure 1B). The genotyping validation of the 140 markers confirmed 74 (67%) polymorphic loci and indentified 37 monomorphic loci, thus classifiable as false positives. Ten (6.8%) SNPs arranged in the Sequenom arrays completely failed in the amplification, whereas 19 (12.9%) had a success amplification rate lower than 70% (Additional file 6). We discarded these 29 SNPs as they did not meet the quality threshold. The majority of the total SNPs (75.5%) amplified successfully. The final number of polymorphic loci corresponded to 67% of the total loci initially designed (Additional file 6). We studied the clusters from which the SNPs were designed, trying to identify the cause for failed SNP genotyping. The *a posteriori *analysis of the sequence clusters indicates that polymorphism in the SNP probe-flanking regions is a probable cause of genotyping failure. The flanking regions of the SNPs failing in the amplification show a higher polymorphism (>3 polymorphisms) than successfully amplifying SNPs (Additional file 7). The design of degenerate SNP probes may alleviate this problem. No other evident difference could be observed in the cluster of sequences used to design SNPs. The average number of overlapping contigs in the clusters, the average length of the contigs and the number of sequences showing the minor allele did not differ between the sequences of the successful and failing SNPs (Additional file 7). We cannot exclude that allele specific amplification due to the proximity of some SNPs to intron/exon boundaries occurred.

### Population genetic structure using neutral SNPs

**Figure 2:**
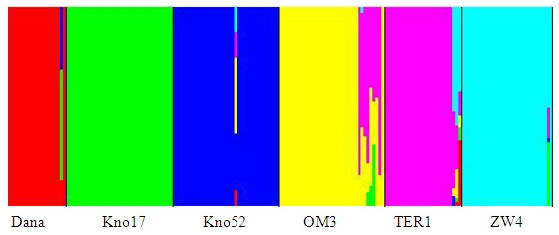
**Population genetic structure based on neutral SNPs**. Non-spatial inference of population genetic structure obtained with the program BAPS [61-63] based on clusters of individuals (populations) and on strictly neutral SNP loci.

We identified 62 strictly neutral SNPs. These markers were used to infer population genetic structure and population demography. The markers did not show a consistent pattern of linkage disequilibrium, and were therefore treated as independent markers. Basic population genetic statistics are described in Additional file 8. The average F_ST _value over all loci was 0.13. F_ST _within inland populations was 0.16, and within coastal populations was 0.11, suggesting a somewhat higher homogeneity in the gene pool of the coastal populations. Pairwise F_ST _were significant for all the pairwise population comparisons (Table 1). There was no significant isolation by distance in the populations studied (r = 0.032; P = 0.44). The non-spatial inference of population genetic structure conducted with BAPS [61,62] clearly identified six genetic units out of six populations (Figure 2). The genetic groups correspond entirely to the discrete populations sampled. We observe a more pronounced admixture in the inland ponds, whereas the ponds along the Belgian coast showed a more uniform gene pool.

**Table 1:** Pairwise F_ST _values among six natural populations of *D*. *magna*

	Dana	Kno17	Kno52	OM3	Ter1	ZW4
Dana	-	*	*	*	*	*
Kno17	0.064	-	*	*	*	*
Kno52	0.084	0.106	-	*	*	*
OM3	0.110	0.103	0.079	-	*	*
Ter1	0.181	0.202	0.197	0.183	-	*
ZW4	0.068	0.159	0.132	0.131	0.155	-

Pairwise F_ST _between all pairs of populations. The lower triangular matrix shows F_ST _values, while the upper triangular matrix shows significant tests (P < 0.05) after Bonferroni correction (*). The geographic location of the six populations as well as other abiotic characteristics are shown in Additional file 2.

The 12 loci that departed from neutral expectation showing reduced polymorphism were present within ESTs. These EST showed similarity mostly to transmembrane proteins (Additional file 9). Four of the 12 loci were linked to biotic stress in either *Daphnia magna *or *D. pulex*. The genomic regions containing the outlier loci will be object of further analysis to indentify functional polymorphism linked to specific environmental stressors.

## Discussion

### EST linked SNPs

SNPs are rapidly becoming the marker of choice for many applications in population ecology, evolution and conservation genetics, because of the potential for high genotyping efficiency, data quality, genome-wide coverage and analytical simplicity (e.g. in modeling mutational dynamics, [2]). However, the use of SNPs in population genetics and genomics is proceeding slower for non-model organisms than for model organisms. One reason is the need of genomics resources for the focal species in order to be able to develop a large panel of SNPs. The second reason is the ascertainment bias due to a limited number of individuals from which the SNP panel is generally developed. In this study we capitalize on rapidly developing genomics resources for *Daphnia *and produce a first set of SNP markers for population genomic studies. The limitations associated with ascertainment bias are alleviated by using individuals originating from different geographic locations and by using different EST libraries (ESTs produced in this study and ESTs retrieved from NCBI GenBank) from which the SNPs are developed.

In comparison to SNPs developed from genomic sequences, EST-derived SNPs have several advantages [65-67]. Since ESTs are transcribed sequences, EST-derived SNPs are associated with actual genes allowing the use of gene-associated SNPs for mapping and comparative genome studies ([68] and references therein). EST-derived SNPs are also a rich source of candidate polymorphisms underlying important traits leading to the identification of quantitative trait nucleotides (QTN) (e.g. [69]) linked to ecologically relevant genes. With this study we have achieved a first step toward the identification of candidate point mutations that can be linked to phenotypic responses to environmental stress in future experimental studies.

### Validation of SNPs in population genetic analysis

Our screen for outlier loci identified a large portion (12%) of loci showing reduced polymorphism as compared to neutral expectations and therefore potentially under selection. The proportion of loci showing reduced genetic variation was higher than generally observed in literature (2.6 to 5.5% in humans [70]; 1.4-3.2% in lake whitefish ecotypes [71]; 2.6 to 3.3.% in Norway spruce [72]; 5.5% in white spruce [73]; 9.5% in salmon [74]; 1.3-3.6% in common frog [75]; 5% in sticklebacks [76]). Most of the mentioned studies are based on anonymous markers or markers falling outside genic regions, whereas our markers are designed within expressed genomic regions. This is likely to have increased the percentage of loci potentially under direct effect of selection in our analysis. In addition, the pre-exposure of animals used to generate the EST libraries to stressors known to induce microevolutionary and adaptive responses in *D. magna *may have increased the efficiency in targeting loci under selection. Further studies on the genes where the outlier SNPs are located will be needed to identify candidate genes underlying adaptive responses to environmental stressors.

The analysis of the population structure based on the neutral loci in six natural populations of *D. magna *revealed patterns that are consistent with discrete populations distributed at regional scale. This is consistent with earlier work on population genetics of *Daphnia *populations. However, the level of genetic differentiation among our populations is lower than previously observed in *D. magna *[77,78]. This can be explained by the use of different genetic markers[79]. The use of allozymes [77,78] vs. SNPs (present study) may have contributed to the somewhat larger values of genetic differentiation observed in previous studies compared to the current study. Even though we observe an overall lower genetic differentiation than previously observed, our results conform to the emerging pattern from literature, with significant genetic differentiation among populations even at relatively short geographic distances (e.g. [27,29,80,81]). The use of SNPs in ecology and population genetics is still uncommon [2,3], therefore it is difficult to draw comparisons between the range of genetic differentiation observed in our study and literature studies. However, recent simulation studies on the use of SNPs in ecology [3] illustrate that ~30 SNPs should be sufficient to detect moderate (F_ST _= 0.01) levels of differentiation, while studies aimed at detecting demographic independence (e.g. F_ST _< 0.005) may require 80 or more SNPs. The number of SNPs used in this study is dense for a species with a relatively small genome size (~200Mb). Additionally, the F_ST _values measured in our populations range between 0.06 and 0.20. This conforms to high among-populations genetic differentiation previously observed using other genetic markers [77,78].

We do not observe isolation-by-distance, which is in agreement with other studies at similar regional geographic scales for *D. magna *(e.g. [78]). The absence of isolation-by-distance reflects high genetic differentiation among neighboring populations rather than the absence of genetic differentiation at larger geographic distances. Earlier reports on isolation-by-distance in *Daphnia *refer to an increase in genetic differentiation at near-continental scales (phylogeographic patterns; see e.g.[82]). Furthermore, the absence of isolation by distance coupled with a strong spatial structure of the populations conform to patterns encountered in other metapopulation systems and can be explained with high genetic drift in small populations [81].

## Conclusions

Building upon the rapid development of genomic tools for *D. magna *we produced and validated a first set of SNP markers from EST sequences to use in future population genomic studies. By exposing animals that were used to produce the EST libraries to standardized selection pressures, we identified candidate EST potentially underlying responses to environmental stress. The SNPs developed in the present study represent an important first step toward the identification of candidate genes underpinning stress responses in natural populations.

## Authors' contributions

LO carried out the population genomic studies, performed the data analysis and drafted the manuscript. MJ produced the EST libraries and carried out the gene annotation analysis. ES designed and applied the pipeline for SNP discovery. SG carried out laboratory work for part of the project and acted as technical support for the authors. LDM supervised and coordinated the study, and helped finalizing the manuscript. All authors read and approved the final manuscript.

## Authors' contributions

LO is a postdoctoral researcher at KULeuven. She is a molecular ecologist with expertise in population genetics and functional genomics. MJ is a PhD student at KULeuven (supervisor LDM). Her thesis is on linking trait and gene expression responses to anthropogenic and natural stressors in the waterflea *Daphnia magna*. ES is a postdoctoral researcher in bioinformatics. At the time of the project she was completing her PhD thesis in bioinformatics at KULeuven (supervisor Filip Volckaert). SG was a technician at KULeuven during this project and strongly involved in the development and use of genetic markers. LDM is professor in ecology and evolution at KULeuven. His research programme focuses on responses of populations and communities to environmental gradients, with strong focus on eco-evolutionary dynamics and ecological genomics of *Daphnia*.

## Supplementary Material

Additional file 1 **Description *Daphnia magna *cDNA libraries from NCBI GenBank**. EST sequences and cDNA library types of *D. magna *sequences retrieved from GenBank at the time of the analysis. Click here for file

Additional file 2 **Natural populations of *Daphnia magna *used for SNP validation and their environmental characteristics**. List of populations from Belgium used for SNP validation and their environmental characteristics. N = population size; Fish = presence (1)/absence (0) of fish; Land use = high (1)/low (0) land use intensity; Parasite = presence (1)/absence (0) of the parasite *Pasteuria ramosa*. Sampling date and environmental variables as measured at the sampling sites are also listed. Transparency was measured by means of Secchi disk. Click here for file

Additional file 3 **PCR and oligonucleotide probes used in the Sequenom MassARRAY platform for SNP typing**. List of SNP loci genotyped using the Sequenom MassARRAY platform. The PCR primers, the oligonuocletide probes and the multiplex information are shown. The sequences of the SNP flanking regions have been deposited in NCBI dbSNP. Click here for file

Additional file 4 **Summary of the gene annotation of the EST sequences**. In this file we report the gene annotation for three set of sequences based on BLAST searches in NCBI and in the *Daphnia *portal (http://wfleabase.org/), called wfleabase in the remaining text): 1) ESTs generated for this study exposing animals to three key environmental stressors and using suppressive subtractive hybridization. The results for this set of sequences are summarized in the spreadsheets EST_1070_NCBI and EST_1070_wfleabase_aa. In EST_1070_NCBI we summarize the gene annotation results obtained from BLAST searches in the NCBI non-redundant protein database using the program tblastx. In EST_1070_wfleabase_aa we summarize the results obtained from BLAST searches in the non-redundant protein database of the *Daphnia *portal (wfleabase) using the program tblastx. 2) Contigs obtained by assembling EST sequences produced in this study (see point 1 above) and sequences of *Daphnia magna *downloaded from NCBI GenBank at the time of the analysis. The results for this set of sequences are summarized in the spreadsheets Contigs_NCBI_1812, Contigs_wfleabase_aa_1812, and Contigs_wfleabase_na_1812. In Contigs_NCBI_1812 we summarize the gene annotation results obtained from BLAST searches in the NCBI non-redundant protein database using the program tblastx. In Contigs_wfleabase_aa_1812, and Contigs_wfleabase_na_1812 we summarize the results obtained from BLAST searches in the non-redundant protein database and in the nucleotide database of the *Daphnia *portal (wfleabase) using the programs tblastx and tblastn, respectively. 3) Contigs obtained from clusters of sequences mined for SNP markers. The number of contigs mined for SNPs is lower than the total number of contigs including our sequences and sequences from GenBank (point 2 above) as several stringent criteria were adopted to select them (see Methods). The results for this set of sequences are summarized in the spreadsheets Contigs_NCBI_574, Contigs_wfleabase_aa_574, and Contigs_wfleabase_na_574. Results from BLAST searches were obtained as in point 2 of this table legend. Columns ID in the described spreadsheets are as follows: 1) SID: sequence identity; 2) GOID - Gene ontology term identity; 3) PID - Protein identity as from BLAST searches; 4) P_desc - Gene description as from BLAST searches and indication of the species where it was identified; 5) e-value - significant homology between the sequence query and the hit in NCBI; 6) Paralog - the paralog group identity (several members may be shown); 7) Start-End: FrameFS - open reading frames predictor results with indication of the start and end coordinates and the frame; 8) DomainID:desc - protein site scan domain identity and description of the protein domain; 9) length - length of the EST; 10) OG_ID - group identity of the ortholog group of protein sequences. This analysis is based on searches for orthologs in several genomes; 11) E-value - significant homology to the ortholog group of protein sequences; 12) Score - score for the ortholog group of protein sequences analysis. The columns ID from 1 to 12 can be found in the spreadsheets: EST_1070_NCBI, Contigs_NCBI_1812, and Contigs_NCBI_574. In the remaining spreadsheets the following columns ID are present: 1) query id - query identity; 2) database sequence (subject) id - sequence identity in wfleabase; 3) gene id - gene identity in wfleabase; 4) percent identity - percentage of identity between query and the gene in wfleabase; 5) alignment length - match in bp between the query and the gene in wfleabase; 6) number of mismatches - number of mismatches between the query and the gene in wfleabase; 7) number of gap openings - gap openings between the query and the gene in wfleabase; 8) query start; 9) query end; 10) subject start - database sequence (subject) start; 11) subject end - database sequence (subject) end; 12) Expect value-E-value of the match between the query and the subject; 13) HSP bit score - blastp e-value score; 14) Gene_ID - gene identity in wfleabase; 15) Gname - gene name; 16) Gnomon - gene prediction in NCBI; 17) Paralog; 18) Paralog,# - number of paralogs identified; 19) OrthoID - ortholog identity; 20) ArpGene - homology to the arthropod genes list; 21) ArpDE - arthropod genes description; 22) Scaffold - scaffold number where the query was annotated; 23) Begin - query start on the scaffold; 24) End - query end on the scaffold; 25) Or - orphan gene; 26) KOG_JGI - ortholog and paralog proteins identities provided for a JGI-sequenced organism; 27) KOG_EMBL - ortholog and paralog proteins identities provided in the EMBL database; 28) meNOG_EMBL - evolutionary genealogy of genes; 29) Enzyme_JGI - protein identity reported in JGI; 30) Enzyme_JGI - protein identity reported in EMBL; 31) Description_JGI - protein description based on JGI database; 32) GeneOntology_JGI - Gene ontology as described in the JGI database; 33) Tandem_ID - identity of tandem genes arrangements. The columns ID are listed in the column_IDs spreadsheet. Click here for file

Additional file 5 **Blast hits results based on the NCBI non-redundant protein database**. List of species whose sequences showed significant homology to the EST sequences from *Daphnia magna*, based on similarities by BLAST searches in the NCBI non-redundant protein database. For each species the number of hits found is listed in the second column of the table. In total, 651 of the 685 EST sequences showed homology to sequences in other species. The list of different genes identified in the dataset ('genes'), the redundancy of the identified genes ('genes redundancy') and the number of times in which each gene was found in different species ('redundancy in species') are also shown. Click here for file

Additional file 6 **List of the EST-linked SNP and descriptive statistics**. List of SNP markers in the set of 147 SNP targeted for genotyping with the Sequenom MassARRAY platform. The protein changes both at the synonymous (S) and the non-synonymous (NS) sites, the codon position of the point mutation, the genotyping success rate, and the minor allele frequency are shown. The characteristics of the contigs from where the SNPs were developed are also shown, in terms of length, polymorphism, and number of sequences in the contig. Nr: SNPs that did not fit in any assay design. Click here for file

Additional file 7 **Features of the EST contigs from which SNP markers were developed**. Main features of the EST contigs from which SNP markers were designed. The contigs of the SNPs that failed in the genotyping process and the ones with a success rate larger than 70% are shown. Click here for file

Additional file 8 **Population genetic statistics in the six natural populations used for the SNPs validation**. Population genetic statistics in the set of six populations used to validate the SNP markers. Ho = observed heterozigosity; He = expected heterozigosity, frequency of the two SNP alleles in the population, H-W = Hardy-Weinberg disequilibrium test (P < 0.05). Click here for file

Additional file 9 **Gene function of the contigs where SNP outliers were detected**. List of outlier loci and the corresponding EST sequences with accession numbers to NCBI GenBank from which the SNPs were developed. The gene function was inferred from the EST contigs. Click here for file
